# Hepatitis C Virus Infection of Cultured Human Hepatoma Cells Causes Apoptosis and Pyroptosis in Both Infected and Bystander Cells

**DOI:** 10.1038/srep37433

**Published:** 2016-12-15

**Authors:** H. M. Kofahi, N. G. A. Taylor, K. Hirasawa, M. D. Grant, R. S. Russell

**Affiliations:** 1 Division of Biomedical Sciences, Faculty of Medicine, Memorial University, St. John’s, Newfoundland A1B 3V6, Canada

## Abstract

Individuals infected with hepatitis C virus (HCV) are at high risk of developing progressive liver disease, including cirrhosis and hepatocellular carcinoma (HCC). How HCV infection causes liver destruction has been of significant interest for many years, and apoptosis has been proposed as one operative mechanism. In this study, we employed a tissue culture-adapted strain of HCV (JFH1_T_) to test effects of HCV infection on induction of programmed cell death (PCD) in Huh-7.5 cells. We found that HCV infection reduced the proliferation rate and induced caspase-3-mediated apoptosis in the infected cell population. However, in addition to apoptosis, we also observed infected cells undergoing caspase-1-mediated pyroptosis, which was induced by NLRP3 inflammasome activation. By co-culturing HCV-infected Huh-7.5 cells with an HCV-non-permissive cell line, we also demonstrated induction of both apoptosis and pyroptosis in uninfected cells. Bystander apoptosis, but not bystander pyroptosis, required cell-cell contact between infected and bystander cells. In summary, these findings provide new information on mechanisms of cell death in response to HCV infection. The observation that both apoptosis and pyroptosis can be induced in bystander cells extends our understanding of HCV-induced pathogenesis in the liver.

Hepatitis C virus (HCV) infection continues to be one of the major health challenges in the modern world. An estimated 185 million people are infected globally, which constitutes approximately 3% of the world’s population^1^. Access to new HCV treatment remains limited, and in untreated individuals, HCV infection progresses to chronicity in 70–85% of new cases, putting those chronically infected patients at risk of developing severe liver disease, including fibrosis, cirrhosis, and hepatocellular carcinoma (HCC)^2^,^3^. The mechanisms by which these HCV-associated liver diseases develop are poorly understood, but evidence suggests that induction of programmed cell death (PCD) in the HCV-infected liver plays a role in this pathogenic process.

Apoptosis is a non-inflammatory form of PCD that can be induced by either extrinsic or intrinsic pathways. The extrinsic pathway is initiated by the interaction between a cell surface death receptor and its ligand. This interaction results in recruitment of caspase-8 to the cytoplasmic domain of the receptor, leading to their cleavage and activation (reviewed in ref. 4). Once activated, caspase-8 cleaves and activates the executioner caspases (reviewed in ref. 5). This signal can also be amplified by the caspase-8-dependent cleavage of the pro-apoptotic Bcl-2 family member Bid, which then translocates to the mitochondrial membrane to activate the intrinsic apoptotic pathway^6^. The intrinsic pathway can also be initiated by stimuli such as radiation, hypoxia, viral infections, or by the withdrawal of essential growth factors. These stimuli initiate a series of events that induce mitochondrial outer membrane permeabilization and cause release of cytochrome c (cyt c) and other apoptotic factors from the intermembranous space of the mitochondria into the cytosol (reviewed in refs 7 and 8). Once in the cytosol, cyt c interacts with a protein known as apoptotic protease activating factor-1 (APAF-1), inducing its oligomerization to form a wheel-like structure of seven APAF-1 molecules known as the apoptosome. The apoptosome then binds and activates caspase-9, the initiator caspase for the intrinsic pathway, which in turn cleaves and activates the executioner caspases (reviewed in ref. 9). Apoptotic cells display a number of characteristic features, including plasma membrane budding, apoptotic body formation and DNA fragmentation (reviewed in refs 5, 10 and 11).

Pyroptosis is a caspase-1-mediated, pro-inflammatory form of PCD^12^. It is initiated by a group of cytosolic sensors that belong to the NLR or HIN-200 receptor families (reviewed in ref. 13). Upon stimulation, these receptors self-oligomerize and recruit other proteins to form a multiprotein complex known as the inflammasome^14^. The inflammasomes then act as platforms for caspase-1 activation and maturation of the inflammatory cytokines IL-1β and IL-18^14^,^15^. Activation of caspase-1 results in pyroptosis, which lyses the cell and releases its contents into the extracellular environment. Pyroptosis also shares certain features with apoptosis, such as DNA fragmentation^16^.

Induction of different forms of PCD by HCV infection is believed to be one of the factors that contributes to development of progressive liver disease. Apoptosis of hepatocytes and engulfment of apoptotic bodies by hepatic stellate cells and resident macrophages was found to activate hepatic stellate cells to release TGF-β, thereby hastening the process of fibrosis^17^,^18^,^19^,^20^. Furthermore, TGF-β induces a biological process known as epithelial-mesenchymal transition (EMT) in hepatocytes^21^. EMT participates in progression of many types of cancer, including hepatocellular carcinoma (HCC) (reviewed in ref. 22). The pro-inflammatory nature of pyroptosis suggests that this form of cell death could contribute to the chronic inflammation and pathogenesis associated with HCV infection. The release of danger-associated molecular patterns (DAMPs) from lysed pyroptotic cells can recruit immune cells and further promote inflammation^23^. Activated inflammatory cells in the liver contributes to generation of a pro-carcinogenic environment though production of reactive oxygen species (ROS) and reactive nitrogen species, and the peroxidation of lipids^24^. Activation of the NF-κB pathway, which is a hallmark of inflammatory response, can be involved in fibrogenesis as well as in initiation and progression of HCC in the chronically infected liver (reviewed in ref. 25). Several other reports described an association between the degree of liver inflammation and development of HCC^26^,^27^,^28^. In addition, inflammation and the release of ROS, inflammatory cytokines and chemokines by Kupffer cells are believed to activate hepatic stellate cells, thereby promoting liver fibrosis (reviewed in refs 29 and 30).

In this study, we tested the effect of HCV infection on induction of PCD. We demonstrated that HCV infection induced two distinct forms of PCD, both apoptosis and pyroptosis. Furthermore, we found that these two forms of PCD were induced not only in infected cells, but also in uninfected cells, *i.e.*, bystander apoptosis/pyroptosis. We report here that bystander apoptosis, but not bystander pyroptosis, is cell-cell contact-dependent. These results contribute towards understanding the mechanisms underlying for the development of HCV-associated progressive liver disease.

## Results

### Hepatitis C virus infection reduced cell viability and proliferation, and induced programmed cell death

**Figure 1:**
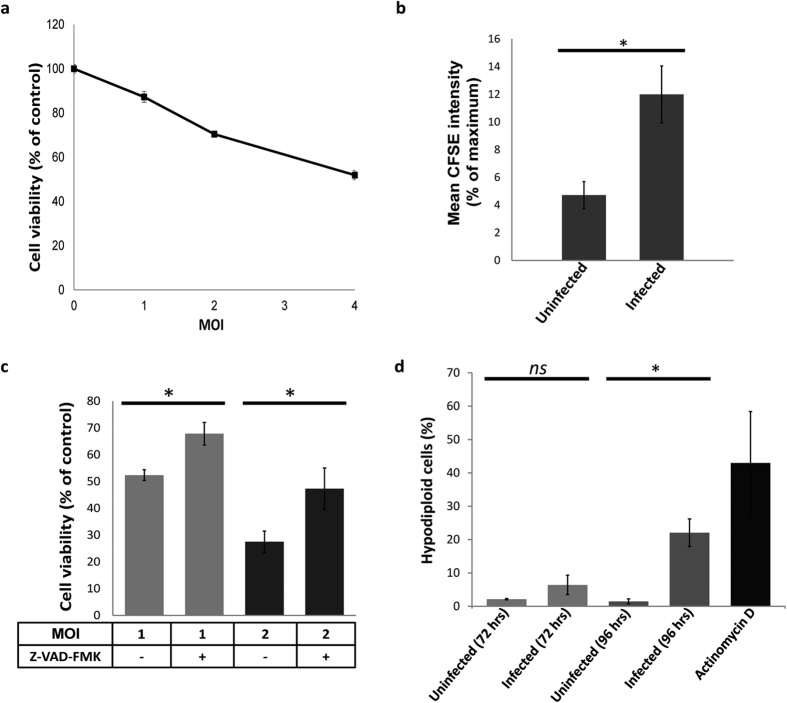
Cell viability and cell cycle analysis in the context of HCV infection. (**a**) Huh-7.5 cells were infected with JFH1_T_ at MOI of 1, 2 or 4. Three days p.i. the total viability of the cells was tested by MTT assay. This data is representative of three independent experiments. (**b**) Infected or uninfected Huh-7.5 cells were labelled with CFSE stain and CFSE intensity was measured 4 days p.i. The data is presented as the mean percentage of CFSE intensity compared to the maximum intensity measured immediately after infection. The data shown is the average from three independent experiments with error bars indicating SD. (**c**) Huh-7.5 cells were infected at MOIs of 1 or 2, then incubated for 4 days in a complete medium containing either 20 μM Z-VAD-FMK or an equivalent volume of DMSO. Cell viability was measured on day 4 p.i. by MTT assay. Data shown is representative of two independent experiments in which each sample was tested in triplicate. The data is presented as the percentage of the viability compared to the uninfected control. (**d**) Infected or uninfected Huh-7.5 cells were harvested 72 or 96 hrs post-infection. Huh-7.5 cells incubated for 48 hrs in complete media containing 50 ng/ml of actinomycin D were used as a positive control. Harvested cells were permeabilized and stained with PI, then cell cycle analysis was performed and the percentage of hypodiploid cells was determined. The data is presented as the mean percentage of the hypodiploid cells from four independent experiments +/−SD., *p* < 0.05 indicated by an asterisk (Student’s t-test).

We first tested the effect of HCV infection on viability of the infected cell population *in vitro*. This was achieved by infecting Huh-7.5 cells with JFH1_T_ at different MOIs and assessing cell viability 3 days p.i. by MTT assay. Infection significantly reduced the total number of viable cells (Fig. 1a). Higher MOIs caused greater reductions in viability. These lower levels of total cellular viability could result from either a reduction in the proliferation rate or increased cell death in the infected cell population. Previous reports have shown that HCV infection reduces proliferation rate^31^,^32^,^33^,^34^. To confirm this effect in our system, we compared the proliferation rate of uninfected versus infected cells using the CFSE dilution assay. The HCV-infected cell population had a significantly higher mean CFSE intensity on day 4 p.i. compared to the uninfected cell population (Fig. 1b). In agreement with previously published reports, these results confirmed that HCV infection reduces cellular proliferation^31^,^32^,^33^,^34^.

One possible mechanism that could explain the effects of HCV infection on cell viability is induction of programmed cell death. Apoptosis and pyroptosis are two types of PCD that are both dependent on activation of caspases. To test the possibility that the reduction in viability was caused by induction of one or both of these forms of PCD, we tested the effect of blocking the activation of caspases on the total viability of the HCV-infected Huh-7.5 cell population. Infected (MOIs of 1 and 2) or uninfected control cells were treated with the pan-caspase inhibitor Z-VAD-FMK. Inhibition of caspases by Z-VAD-FMK increased the total viability of the HCV-infected population of cells (Fig. 1c). This result indicated that caspase-dependent PCD was induced and contributed to the reduction in viability observed in response to HCV infection. We also tested the effect of HCV infection on DNA fragmentation, a feature shared by both apoptosis and pyroptosis^16^,^35^,^36^,^37^. DNA fragmentation results in the formation of low molecular weight DNA fragments that can be extracted from fixed/permeabilized cells, leaving these cells with less DNA than a normal cell. Upon cell cycle analysis, cells undergoing apoptosis/pyroptosis can be detected as hypodiploid cells^38^. To test for DNA fragmentation, infected or uninfected cells were permeabilized and stained with propidium iodide (PI). Slightly higher levels of hypodiploid cells were detected in the infected cell population on day 3 p.i., and this difference reached statistical significance by day 4 (Fig. 1d). These results confirm that HCV infection induces caspase-dependent, DNA-fragmenting programmed cell death in our infectious culture system.

### HCV infection induces apoptosis

**Figure 2:**
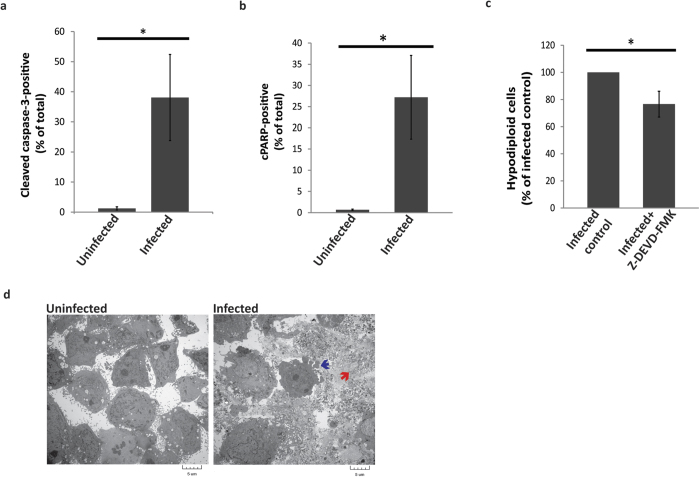
Analysis of apoptosis in HCV-infected cells. Huh-7.5 cells were infected at MOI = 1 and harvested on day 4 post-infection. The harvested cells were stained for cleaved caspase-3 (**a**) or cleaved PARP (**b**). In (**c**) infected cells were cultured in medium containing 100 μM Z-DEVD-FMK or an equivalent volume of DMSO for 4 days, then harvested and stained with PI. Cell cycle analysis was used to determine the number of hypodiploid cells. Data presented in A, B & C are the mean of three independent experiments +/−SD. Data shown in C is the percentage of hypodiploid cells compared to the untreated control. *p* < 0.05 indicated by an asterisk (Student’s t-test). (**d**) EM images of control or infected Huh-7.5 cells showing apoptotic features such as plasma membrane blebbing indicated by the blue arrow. The red arrow indicates cellular debris from a cell that had undergone lysis.

To determine whether the caspase-dependent programmed cell death observed here in the context of HCV infection is due to apoptosis versus pyroptosis, we first performed apoptosis-specific analyses on virus-infected cells. One of the main characteristics differentiating apoptosis from pyroptosis is the type of caspase responsible for their induction. Regardless of the pathway that initiates apoptosis, caspase-3 and other executioner caspases are always activated in order to induce the final events that cause the demise of the cell (reviewed in ref. 5). Pyroptosis, on the other hand, is a caspase-3-independent form of PCD that depends solely on the activation of caspase-1 (reviewed in ref. 39). Caspase-3 activation in the infected cell population was tested by measuring the cleavage of caspase-3 and its substrate Poly (ADP-ribose) polymerase (PARP) by staining the cells with antibodies specific to the cleaved forms of caspase-3 and PARP. We found that HCV infection induced the activation of caspase-3 as significantly higher percentages of cleaved caspase-3- and cleaved PARP (cPARP)-positive cells were detected in the infected versus the uninfected cell population (Fig. 2a,b).

To confirm the role of caspase-3 in the induction of PCD, we treated the infected cell population with a caspase-3-specific inhibitor and monitored the effect of this treatment on the proportion of hypodiploid cells within the target cell population. We found that blockade of caspase-3 caused a small, but significant reduction (~25%) in the percentage of hypodipoid cells contained within the target cell population (Fig. 2c). In agreement with previous studies, these results confirm that HCV infection induces apoptosis in infected cell populations, but the partial (~25%) reduction in cell death resulting from inhibition of caspase-3 suggested that at least one other mechanism of cell death was at work within the target cell population^32^,^40^,^41^,^42^,^43^. We also observed features of apoptosis in the target cell population by electron microscopy as cell condensation and plasma membrane blebbing were clearly evident (Fig. 2d; blue arrow). However, plasma membrane disruption and cellular debris were also observed, indicative of a lytic form of cell death (Fig. 2d; red arrow). These observations, in combination with the relatively moderate effect of inhibiting caspase-3 on reducing the number of hypodiploid cells prompted us to question whether HCV infection also causes pyroptosis, which is known to cause DNA fragmentation, but with lysis of affected cells^16^,^44^.

### HCV infection induces pyroptosis

**Figure 3:**
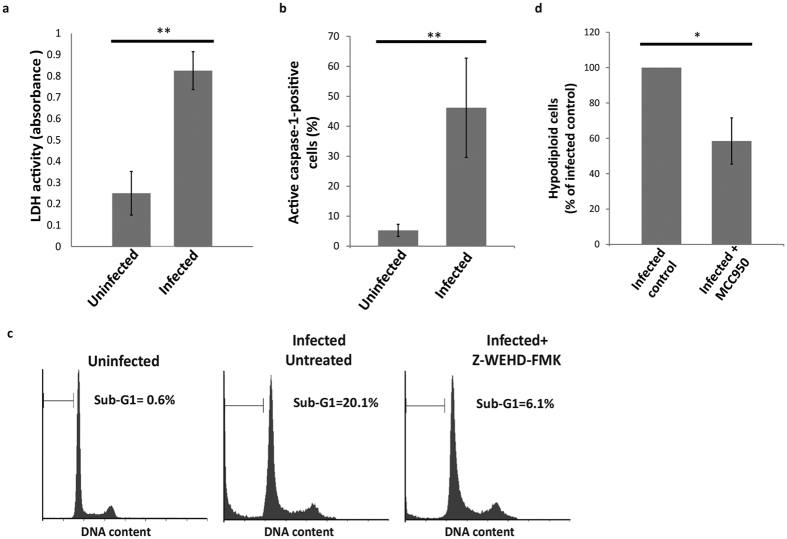
Analysis of pyroptosis in the context of HCV infection. Huh-7.5 cells were infected at MOI = 1 or uninfected. (**a**) Culture fluids were collected four days p.i. and LDH activity was measured. Each bar represents the mean absorbance value from three independent experiments (each measured in triplicate) +/−SD. (**b**) On day 4 p.i., cells were harvested and stained with FAM-YVAD-FMK FLICA, then analysed by flow cytometry to detect cells containing active caspase-1. Six independent experiments were performed and each bar represents the mean percentage of active caspase-1-positive cells +/−SD. (**c**) Infected or uninfected Huh-7.5 cells were incubated in complete medium containing 100 μM Z-WEHD-FMK or an equivalent volume of DMSO. The cells were harvested on day 4 p.i. and stained with PI. Cell cycle analysis was used to determine the percentage of hypodiploid cells (Sub-G1) in each of the populations. Results shown represent two independent experiments. (**d**) Infected or uninfected Huh-7.5 cells were incubated in complete medium containing 0.1 μM MCC950. On day 4 p.i., cells were harvested and stained with PI. Cell cycle analysis was performed to determine the percentage of hypodiploid cells in each of the populations. The data is presented as the mean percentage of hypodiploid cells compared to the infected untreated control. Bars represent the mean of three independent experiments +/−SD. *Indicates *p* < 0.05 and **indicates *p* < 0.005 (Student’s t-test).

We asked then whether HCV infection causes the induction of pyroptosis as a second form of PCD. One of the main morphological features of pyroptosis is loss of the integrity of the plasma membrane, which leads to cell lysis and the release of cellular contents to the surroundings^16^,^44^. Lactate dehydrogenase (LDH) is an enzyme that is normally sequestered inside the cell and is released extracellularly only in the event of cell lysis^45^. Measuring the activity of LDH in the medium of the cultured cells is a useful tool for detecting lytic cell death, including pyroptosis^45^. By measuring the activity of LDH in the medium of HCV-infected or uninfected Huh-7.5 cells, we found that HCV infection resulted in a significant increase of the LDH activity in the medium of infected Huh-7.5 cells (Fig. 3a). It is notable that the LDH release assay is not specific for pyroptosis. Other forms of lytic cell death, such as necrosis and necroptosis, can also result in an increase in the levels of LDH^46^. Moreover, during the late stages of apoptosis, and in the absence of scavenger cells, apoptotic cells undergo secondary necrosis, which also releases the cellular contents, including LDH, to the exterior of the cell^47^. Despite the lack of specificity for detecting pyroptosis, LDH release is one of the predominant characteristics of cells undergoing pyroptosis, thereby supporting our theory that HCV infection induces pyroptosis. Interestingly, upon re-examination of EM images representing cells infected with HCV, lysed cells that may have died by pyroptosis can be observed (Fig. 2d; red arrow).

By definition, pyroptosis is a caspase-1-dependent form of PCD, and this requirement distinguishes pyroptosis from apoptosis^12^. To test whether HCV infection could induce pyroptosis, we stained virus-infected cells with FAM-YVAD-FMK FLICA reagent, which specifically recognizes the active form of caspase-1. We found that HCV infection caused a significant increase in the proportion of active caspase-1-postive cells (~45% compared to ~5%; Fig. 3b). This high level of caspase-1 activation in combination with increased levels of LDH and the EM observations described above strongly suggest that HCV infection induces pyroptosis.

To further confirm the induction of pyroptosis by HCV infection, we tested whether this PCD could be specifically blocked by caspase-1 inhibition. This was done by treating the HCV-infected or control cells with the caspase-1-specific inhibitor Z-WEHD-FMK, and measuring the effect on the number of hypodiploid cells. Inhibiting caspase-1 rescued more than half of the cells undergoing HCV-induced PCD (Fig. 3c), confirming that pyroptosis is induced in the HCV-infected cell population.

One of the major functions of the active form of caspase-1 is to induce the cleavage and maturation of IL-1β, a pro-inflammatory cytokine^48^. We tested this function by measuring the levels of IL-1β in the supernatant of the infected cells by ELISA. Surprisingly, we could not detect IL-1β in the supernatant of the infected cells (data not shown).

Caspase-1 can be activated by different types of inflammasomes depending on the nature of the stimuli that induce their activation. Viral RNA is known to induce the assembly and activation of NLRP3 inflammasomes^39^. This prompted us to ask whether HCV-induced pyroptosis is stimulated through NLRP3 inflammasome activation. To address this question we treated HCV-infected Huh-7.5 cells with a specific inhibitor for NLRP3 inflammasome activation and monitored the effect of this treatment on induction of cell death. The inhibitor we used was MCC950, which is a recently developed potent, selective, small molecule inhibitor for NLRP3^49^. As expected, we found that inhibiting NLRP3 resulted in a significant decrease in the number of hypodiploid cells (Fig. 3d). This inhibition confirms the role of NLRP3 inflammasome activation in HCV-induced pyroptosis. Taken together, the demonstration of caspase-1 activation, release of LDH, and blockade of cell death by caspase-1 and NLRP3 inhibitors confirm by multiple methods that HCV infection can cause pyroptosis.

### HCV infection induces bystander apoptosis and bystander pyroptosis

The results described above provide evidence that HCV infection induces apoptosis and pyroptosis in the infected cell population. However, the data presented does not distinguish whether this induction is occurring exclusively in the infected cells, or whether it is also taking place in the neighbouring uninfected cells, *i.e.*, “bystander apoptosis/pyroptosis”. Bystander apoptosis has been observed in other viral infections and is believed to contribute to the pathogenesis of these infections. For example, bystander apoptosis was reported to be an important factor in the pathogenesis of cytomegalovirus retinitis^50^,^51^. It also plays a major role in the CD4+ T cell decline seen in HIV infection (reviewed in ref. 52). Moreover, bystander apoptosis is induced by Ebola infection and causes massive lymphocyte death^53^,^54^. Bystander pyroptosis was also described previously in the context of HIV infection and is believed to play a major role in the depletion of the CD4+ T cells (reviewed in ref. 55). To our knowledge, neither bystander apoptosis nor bystander pyroptosis have been reported in the context of HCV infection.

**Figure 4:**
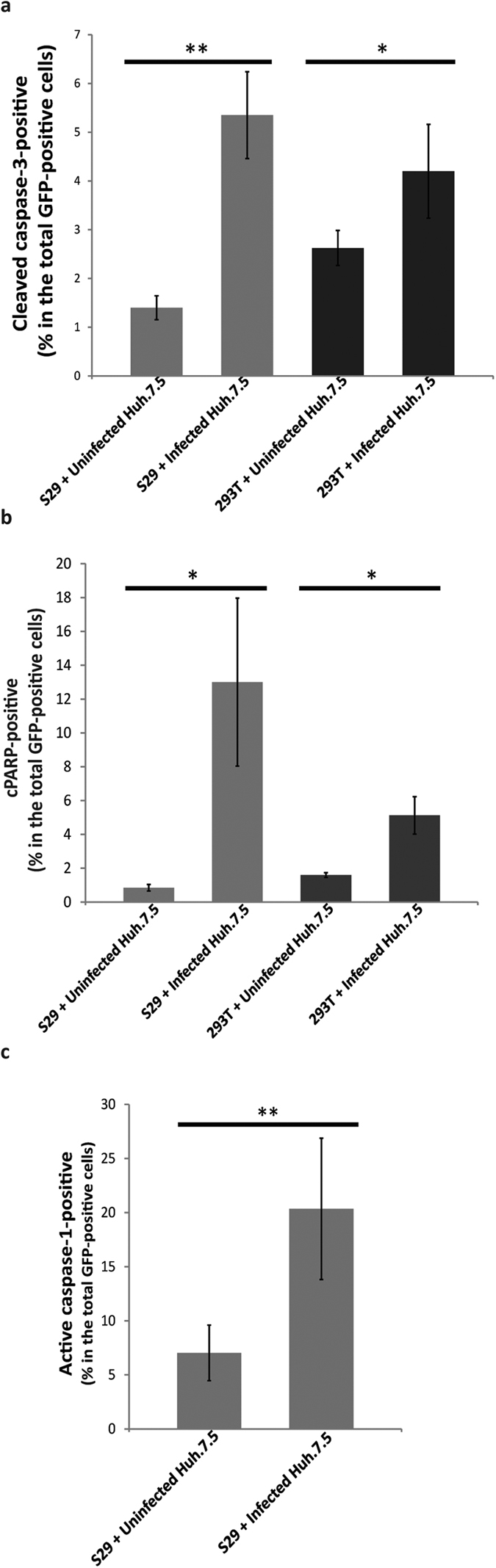
Measurement of bystander apoptosis/pyroptosis. Huh-7.5 cell infected at MOI = 1 or uninfected were co-cultured with either S29 or 293 T cells at a ratio of 5:1. The S29 or 293 T cells were transfected with a GFP-expressing plasmid before the start of the co-culture. On day 4 p.i., cells were harvested and stained with antibodies specific for either cleaved caspase-3 (**a**) or cleaved PARP (**b**), or active caspase-1-specific FAM-YVAD-FMK FLICA (**c**), then analysed by flow cytometry. S29 cells or 293 T cells were identified in the analysis by gating on the GFP positive population. The results are shown as the percentage of cleaved caspase-3, cleaved PARP or active caspase-1-positive cells among the S29 or the 293 T cell populations. Mean +/−SD is shown from at least three independent experiments. *Indicates *p* < 0.05 and **indicates *p* < 0.005 (Student’s t-test).

To investigate the induction of apoptosis and pyroptosis in bystander cells, we employed a strategy in which HCV-non-permissive cells were transfected with GFP and co-cultured with infected or control Huh-7.5 cells. In this system, cell death observed in GFP-positive cells indicates bystander cell death. The HCV-non-permissive cells used in these experiments were the S29 cell line, which is a sub-clone of Huh-7 cells that expressed extremely low levels of CD81, making them 1000-fold less permissive for HCV infection than Huh-7.5 cells^56^. As a control, bystander apoptosis was also investigated in 293 T cells in order to test whether any observed bystander apoptosis was specific for neighbouring hepatocyte-like cells versus other cell types. 293 T cells are a human embryonic kidney cell line that is not permissive to HCV infection due to the lack of claudin-1, which is another important receptor for HCV entry^57^. Very low susceptibility (1000-fold less than Huh-7.5) was reported in these cells and only when they ectopically express claudin-1^57^. We found that co-culturing S29 cells with infected Huh-7.5 resulted in significantly higher numbers of cleaved caspase-3-positive and cleaved PARP-positive cells within the S29 cell population (Fig. 4a,b). Caspase-3 activity was also detected in 293 T cells co-cultured with infected Huh-7.5 cells (Fig. 4a,b), although the degree of induction of bystander apoptosis observed in the 293 T cells was lower than that observed in the S29 cells. These results demonstrated that HCV infection caused the induction of apoptosis in bystander cells, and this effect was not specific to hepatocyte-like cells.

Next we investigated whether pyroptosis can be induced in bystander cells present in the HCV infected population. To do this, S29 cells were co-cultured with infected or uninfected Huh-7.5 cells as above, but the harvested cells were stained with FAM-YVAD-FMK FLICA to detect caspase-1 activity. By gating on GFP-positive cells, we found a significantly high percentage of active-caspase-1-positive cells in the S29 cell population that was co-cultured with infected Huh-7.5 cells, but not when they were co-cultured with uninfected Huh-7.5 cells (Fig. 4c). This observation suggests that the induction of pyroptosis by HCV infection was not limited to the infected cells, but also could be induced in bystander cells. Based on these results, we conclude that HCV-induced pyroptosis and apoptosis occur in both the infected and the bystander cells.

### Cell-cell contact is required for the induction of bystander apoptosis but not for bystander pyroptosis

**Figure 5:**
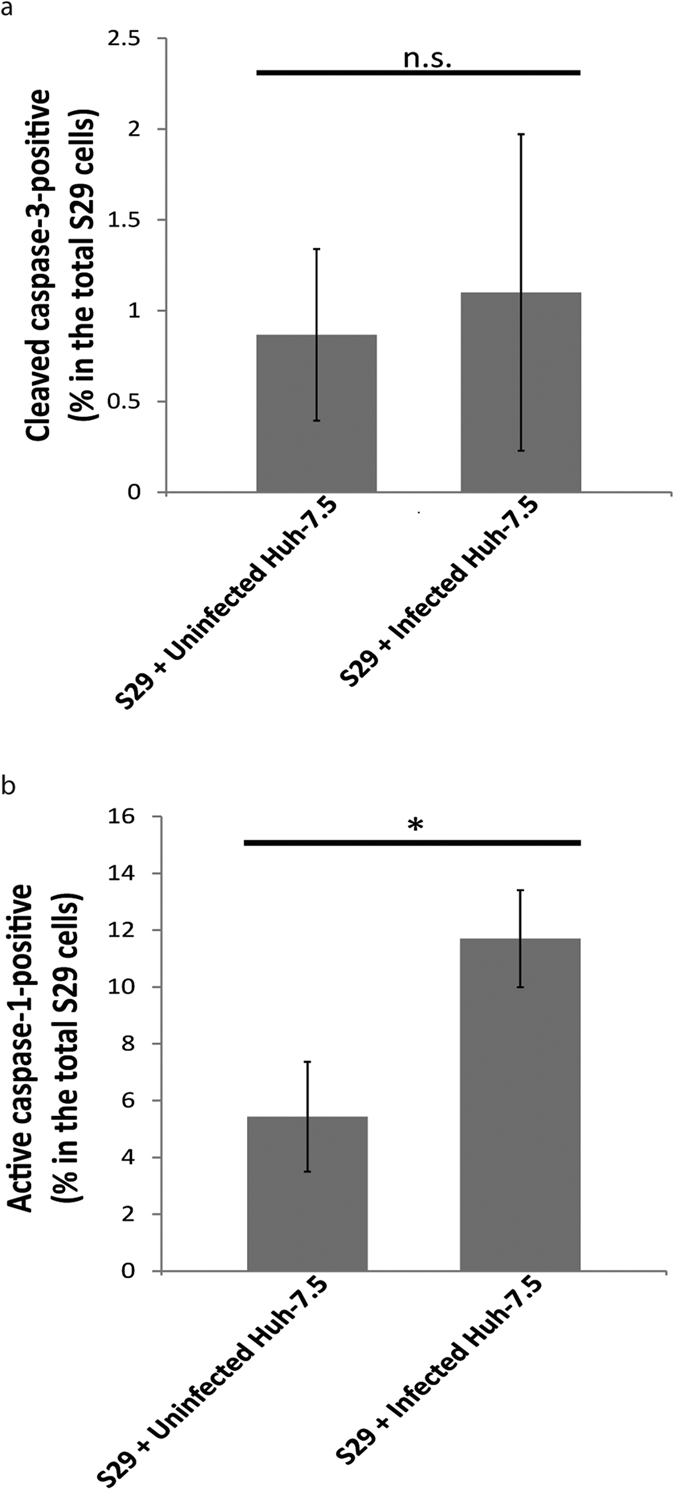
Analysis of contact dependence. Infected or uninfected Huh-7.5 cells were co-cultured with S29 cells in a transwell plate at a ratio of 5:1. Four days p.i., S29 cells were harvested and stained with either cleaved caspase-3 antibody (**a**) or FAM-YVAD-FMK FLICA for active caspase-1 (**b**). The results are represented as the mean percentage +/−SD of cleaved-caspase-3-positive, or active caspase-1-positive cells among the total S29 cells from three independent experiments. *n.s.:* Not significant*, p* < 0.05 indicated by an asterisk (Student’s t-test).

The PCD we observed above in the bystander cells could be induced by two possible mechanisms: a direct interaction between the infected cell and the uninfected bystander cell; or through the production of soluble mediators or exosomes released by the infected cell. To determine which of these mechanisms is responsible for HCV-induced bystander apoptosis/pyroptosis, we performed transwell assays. In these experiments, we prepared a co-culture of infected versus uninfected Huh-7.5 cells (lower chamber) with S29 cells (upper chamber) at the same ratio used in the previous section (5:1). Inserts with a pore diameter of 0.4 μm were chosen to allow the exchange of soluble material and exosomes, but prevent the movement of cells or even apoptotic bodies through them. Following a four day co-culture, the S29 cells were harvested and stained with an antibody specific for cleaved caspase-3 to detect apoptosis or FAM-YVAD-FMK FLICA to detect pyroptosis. Based on the very low levels of cleaved caspase-3-positive cells detected, the S29 cells did not undergo significant levels of apoptosis when co-cultured with infected or uninfected Huh-7.5 cells (Fig. 5a). The lack of induction of apoptosis in the bystander cells, despite the potential for passage of soluble molecules, viruses and exosomes, shows that cell-cell contact is required for the induction of bystander apoptosis. However, a different scenario was observed when we tested the role of cell-cell contact in the induction of bystander pyroptosis. The S29 cells that were co-cultured with infected Huh-7.5 cells were found to have significantly higher percentages of active caspase-1-positive cells than their counterparts co-cultured with uninfected cells (Fig. 5b). This result demonstrates that unlike HCV-induced bystander apoptosis, bystander pyroptosis caused by HCV infection is cell-cell contact-independent.

## Discussion

**Figure 6:**
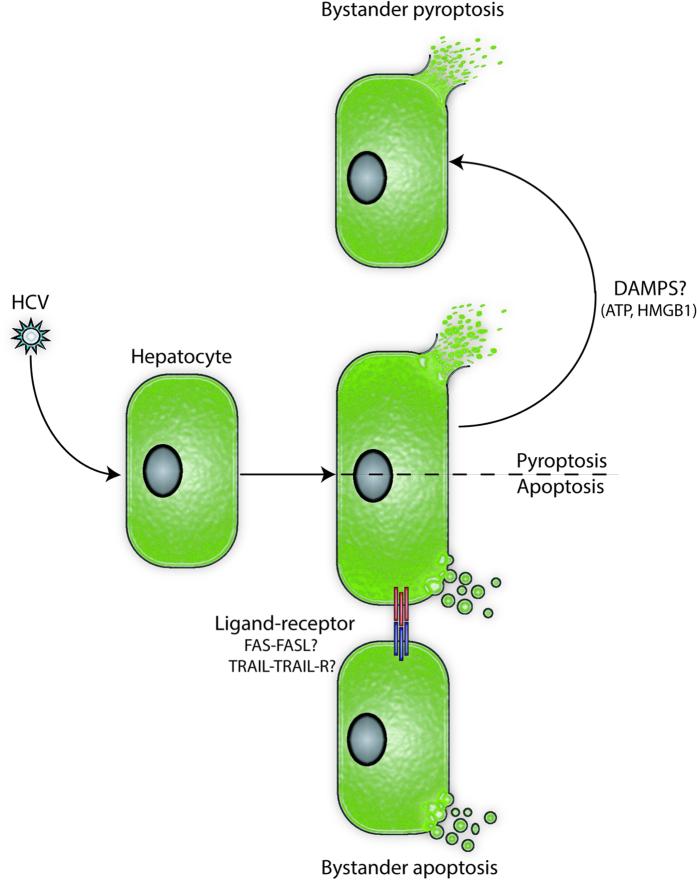
Proposed model for HCV-induced programmed cell death. Schematic representation of the proposed model for the induction of the multiple forms of PCD in response to HCV infection. HCV infection induces at least two forms of programmed cell death in the infected cells: apoptosis and pyroptosis. Apoptosis is also induced in the neighbouring uninfected cells (bystander apoptosis) in a cell-cell contact-dependent manner, presumably by an interaction between a death ligand expressed on the surface of infected cell and a death receptor expressed on the surface of the bystander cell. The induction of bystander pyroptosis is cell-cell contact independent and is mediated by an as yet undetermined soluble factor.

The gradual destruction of the liver that takes place in the context of HCV infection has been a matter of intense interest for many years. In this report, we investigated the possibility that HCV itself causes cytopathic effects in the absence of immune cells. We found that HCV infection indeed induced at least two forms of PCD, those being apoptosis and pyroptosis. We also demonstrated that both infected and neighboring uninfected (bystander) cells were induced to undergo each of these two forms of PCD. The bystander apoptosis we observed, but not the bystander pyroptosis, required direct contact between the infected and uninfected cells. A preliminary model reconciling these results proposes receptor-ligand-mediated apoptosis and soluble factor-mediated pyroptosis (Fig. 6).

Our study employed a cell culture-adapted strain of HCV JFH-1 (JFH1_T_) and human hepatoma-derived Huh-7.5 and related S29 cells. This system is one of the few non-chimeric, highly replicating HCV culture systems that allows a dynamic range of measurement sufficient for studies such as we have performed here. However, cell culture systems such as ours inherently possess caveats and limitations. For example, unlike all other patient isolates of HCV, the JFH-1 strain can propagate autonomously in cell culture without adaptive mutations or modification of target cells. This fact has led many in the field to question whether this strain actually represents natural HCV infection in patients. Additionally, the Huh-7.5 cells used by us and many others are cancer cell-derived and are known to have altered innate immune mechanisms. Given these caveats, any findings such as ours that might have pathogenic implications may not be directly extrapolated to persistent HCV infection of liver and should be confirmed in animal and infected human liver studies.

In a broader sense, it is important to consider whether or not the results presented here have relevance to the disease situation. Given the above-mentioned caveats of HCVcc systems, we cannot say at this point that apoptosis or pyroptosis is actually taking place in infected livers. However, apoptosis has been detected in the human liver cells of SCID/ALB-uPa humanized mice^58^. In human studies, a correlation between apoptotic index and histological grading in liver sections, and activation of caspase-3 and -7, has been demonstrated in liver biopsies from chronically infected individuals^59^,^60^. Based on these studies, it is possible that our results regarding apoptosis are physiologically relevant, but it will be important to revisit this topic in light of our new findings, and more importantly, the topic of pyroptosis has never been addressed in the context of HCV-infected individuals. It would be interesting now to test whether other subtypes/genotypes of HCV cause pyroptosis and bystander cell death because JFH-1 was originally isolated from a patient with fulminant hepatitis^61^. If these forms of PCD are specific to JFH-1 infection, one might speculate that perhaps only highly pathogenic strains of HCV can induce cell death by these mechanisms.

Our results suggest that the induction of bystander apoptosis requires cell-cell contact between the infected and the affected uninfected cells. This mechanism of cell death could take place due to an interaction between death ligands expressed on the infected cell and death receptors present on the bystander cell. The upregulation of death ligands such as Fas-ligand and TRAIL by HCV infection has been reported previously and would make interesting candidates for subsequent studies aimed at identifying the mechanism of bystander apoptosis^41^,^62^,^63^. In this study we showed that activation of caspase-1 induced pyroptosis as blocking caspase-1 activity or the activation of NLRP3 inflammasomes significantly reduced cell death. Despite the clear activation of caspase-1 in infected and bystander cells, we could not detect IL-1β in the supernatant of these cells. Although surprising at first, this result is in agreement with previous reports where it was suggested that hepatocytes do not produce this pro-inflammatory cytokine as a regulatory mechanism to prevent liver toxicity in response to the continuous exposure to blood pathogens^64^,^65^,^66^. As with apoptosis, pyroptosis was not limited to infected cells, but also occurred in neighbouring uninfected cells. However, unlike bystander apoptosis, bystander pyroptosis did not require cell-cell contact. The mechanism of bystander pyroptosis induction is not clear, but lysed pyroptotic cells release a number of DAMPs including: High-mobility group box 1 (HMGB1), heat shock proteins, ATP, DNA and RNA^67^. Many of these DAMPs can induce inflammasome activation in affected cells. For example, extracellular ATP binds to purinergic receptor P2X7 (P2RX7), which opens ion channels leading to K^+^ efflux, resulting in inflammasome activation^67^. HMGB1 stimulates a signaling pathway through TLR4 and receptor for advanced glycation end products (RAGE) that activates NLRP3 inflammasomes and pyroptosis in hepatocytes^68^. Understanding the mechanism by which bystander pyroptosis is taking place in our system would be of significant interest, however, pyroptosis has not yet been demonstrated in HCV-infected individuals, but would certainly be interesting to study.

How HCV can induce two distinct forms of PCD simultaneously remains to be determined. It is possible that different viral components are independently detected by separate sensors, or that different steps of the viral life cycle induce distinct cellular responses ultimately resulting in the independent induction of each form of PCD. Alternatively, perhaps the HCV-induced apoptotic and pyroptotic pathways share a common origin that activates both of them. Interestingly, recent evidence suggests that in addition to their classical role in activating caspase-1 and inducing pyroptosis, NLRP3 inflammasomes can also activate caspase-8, which in turn induces apoptosis^69^,^70^,^71^,^72^,^73^. This could be the case in HCV infection whereby activation of the NLRP3 inflammasome activates caspase-8 and caspase-1 to induce both apoptosis and pyroptosis, respectively. It is also possible that both apoptosis and pyroptosis are induced by the same cellular response to HCV infection. For example, in genetically obese mice, induction of ER stress in hepatocytes was reported to activate NLRP3 and to induce both apoptosis and pyroptosis^74^.

It is not clear at this point whether apoptosis or pyroptosis of infected versus bystander cells is induced by the same mechanisms. Presumably, certain responses, such as ER stress, occur solely in the infected cells, logically causing the infected cells to be more prone to apoptosis/pyroptosis than the bystander cells. Furthermore, the foci of infected cells typically observed in HCVcc-infected Huh-7.5 cells demonstrates that viral infection preferentially spreads to the closely neighbouring cells. This means that most infected cells are in close proximity to other infected cells and could therefore be easily affected by the same stimuli that induce the bystander apoptosis/pyroptosis. Therefore, infected cells would be under higher pressure to undergo apoptosis/pyroptosis than the bystander cells. In support of this idea, regarding both apoptosis and pyroptosis, we observed generally higher levels of cell death in the overall population of cells than when we gated on only the uninfected cells. However, many viruses have evolved strategies for inhibiting cell death in its host in order to allow for the establishment and maintenance of virus replication (reviewed in ref. 75). HCV is no exception; most HCV proteins have been reported to possess anti-apoptotic effects^76^,^77^. We don’t typically detect apoptosis in HCV-infected cells until day four post-infection. So it is possible that HCV somehow initially blocks apoptosis, and if so, this capability would confer a survival advantage to infected cells over bystander cells. However, if this were the case, we would expect to see bystander cell death earlier than day four. At this point we can only hypothesize that the balance between pro- and anti-apoptotic/pyroptotic stimuli determines the fate of the infected/bystander cells.

Induction of PCD in response to viral infection is a well-established mechanism for restricting viral infections. It is widely accepted that various immune cells can induce apoptosis in infected cells, but PCD might also be induced by intracellular innate immune responses. However, the induction of these PCD pathways can be circumvented by numerous inhibitory viral proteins (reviewed in ref. 75). These opposing forces place the host cell into a continuous competition with the infecting virus over control of these cell death pathways. The results described here demonstrate that HCV infection of human hepatoma cells induces two distinct forms of PCD: apoptosis and pyroptosis. If similar events are taking place in the infected liver, the simultaneous induction of multiple forms of PCD in HCV-infected hepatocytes could be one of the strategies used by the host cell to overcome the inhibitory effect of the viral proteins. In this regard, HCV must overcome a higher barrier by inhibiting two different death pathways in order to maintain the survival of its host cell and maintain its replication. Nevertheless, it is clear that HCV is a highly evolved pathogen that is able to maintain control over these cell death pathways until it has completed its life cycle and released new viral progeny to infect *naïve* hepatocytes.

In summary, using a fully infectious HCV culture system, this study confirms the ability of HCV to induce apoptosis, but our results now extend the understanding of HCV pathogenesis with the novel demonstration that HCV can also induce pyroptosis in cultured hepatocyte-like cells, and that both of these forms of programmed cell take place in uninfected bystander cells in the presence of HCV-infected cells. Recently identified direct-acting antiviral agents have shown extremely high cure rates, but HCC can still develop even after the elimination of virus from an infected individual. We believe that understanding the exact mechanisms by which the virus stimulates these two forms of PCD in infected and bystander cells might provide us with targets for the development of novel treatments and eventually a comprehensive therapeutic regimen that can both eliminate virus and prevent progression of HCV-related liver disease in HCV-infected patients.

## Materials and Methods

### Cells

Infection, transfection and co-culture experiments were performed in Huh-7.5 (gift from C. Rice, Rockerfeller University, USA)^78^, S29 (gift from S. Emerson and R. Purcell, National Institutes of Health, USA)^56^ and 293 T (Cedarlane Canada) cells at 37 °C in the presence of 5% CO_2_. All cells were cultured in complete medium (CM) consisting of Dulbecco’s Modified Eagle Medium (DMEM) (Invitrogen) supplemented with 10% fetal calf serum (FCS) (Invitrogen) and 1% penicillin/streptomycin (Invitrogen).

### Virus

For this study, we used JFH1_T_, a tissue culture-adapted strain of JFH-1 containing three adaptive mutations within E2, p7 and NS2^56^,^79^. To generate virus stocks, 1 × 10^6^ Huh-7.5 cells were seeded in 10 cm culture dishes and cultured overnight. The following day, cells were transfected with *in vitro*-transcribed viral RNA representing cell culture-adapted JFH1_T_ using DMRIE-C reagent (Invitrogen) as per the manufacturer’s protocol. Virus-containing medium from transfected cells was collected three days post-transfection and virus titre was determined using a limiting dilution focus-forming assay described below^80^. Titred virus-containing medium was inoculated onto virus-naive Huh-7.5 cells for 3 hr at a multiplicity of infection (MOI) of 0.5. Following inoculation, culture medium was replaced with fresh complete medium and cells were cultured for three days. Culture medium from infected cells was then passaged on naïve Huh-7.5 for an additional round of infection in order to eliminate residual input RNA. Culture fluids were then harvested and clarified through Millex-HV 45 μm filters (Millipore). Virus titre was determined by performing a 10-fold serial dilution of the virus stock followed by infection in 8-well chamber slides that had been seeded with 50,000 Huh-7.5 cells/well on the previous day. Three days post-infection (p.i.), slides were stained with anti-HCV core antibody (B2, Anogen), followed by goat anti-mouse Alex Fluor^®^ 488 (Invitrogen), and the number of foci in the highest positive dilution were counted. From this number the titre was expressed as focus forming units per millilitre (FFU/ml).

### Immunostaining for indirect immunofluorescence

Medium was aspirated from the wells of the 8-chamber slides and cells were washed by immersing the slide in 1X phosphate-buffered saline (pH = 7.4; PBS) for 2 minutes. The cells were then fixed and permeablized by immersing the slide in 100% acetone for 2 minutes. For HCV core staining, the slides were covered with mouse monoclonal anti-HCV core antibody (B2, Anogen) diluted 1:200 in 5% BSA in PBS for 20 minutes. Slides were washed in PBS for 5 minutes, then incubated for 20 minutes with the secondary antibody (goat anti-mouse Alexa Fluor^®^ 488; Invitrogen) diluted 1:100 in PBS. The slides were then washed and mounted with Vectashield Hard Set mounting medium containing DAPI (Vector Laboratories). The slides were examined at 10X and 20X magnifications on a Zeiss Axio Imager.M2 immunofluorescence microscope.

### MTT assay

In a 96-well plate, 5,000 Huh-7.5 cells were seeded in 100 μl of complete medium per well. The next day, cells were infected at different MOIs (1, 2, and 4) by aspirating the medium covering the cells and replacing it with 50 μl of the appropriate virus stock to give the desired MOI. After 4 hours of incubation, the virus inocula were aspirated and replaced with 100 μl of fresh medium. The plates were then incubated at 37 °C for 72 hours until the day of MTT assay. MTT (3-[4,5-dimethylthiazol-2-yl]-2,5 diphenyl tetrazolium bromide) was prepared at 5 mg/ml in PBS and diluted 1:10 in complete medium to generate the MTT working stock. Medium was removed from wells and replaced with 100 μl of MTT working stock, then incubated for 4 hours at 37 °C. Following incubation, liquid was removed from the wells carefully so as not to disturb the formazan crystals that had formed at the bottom of the wells. These crystals were then dissolved by adding 100 μl of DMSO to each well and lightly shaking the plate for 10 minutes. The optical density of the solution in the wells was then read on a plate reader at 540 nm.

### CFSE assay

Huh-7.5 cells were seeded in 10 cm dishes at 1 × 10^6^ cells/dish and infected on the next day at an MOI of 2 for 4 hours. Cells were then harvested and washed with complete medium, then incubated for 15 minutes in pre-warmed (37 °C) solution containing 10 μM Carboxyfluorescein diacetate succinimidyl ester (CFDA-SE) in PBS. The CFDA-SE solution was then removed and replaced by complete medium. Cells were then incubated for 30 minutes at 37 °C and harvested daily on days 0–5. Cells were examined daily by light microscopy to ensure that the cells were not confluent. At the specified time point, cells were harvested, fixed with 2% paraformaldehyde in PBS, and washed with PBS. Finally, the CFSE intensity in the cells was measured by flow cytometer in the FL1-H channel of a Becton Dickinson FACS Calibur.

### Propidium iodide (PI) staining and cell cycle analysis

Huh-7.5 cells were seeded in 10 cm dishes at 1 × 10^6^ cells/dish and infected on the next day at an MOI of 1. The PI staining protocol used here was adapted with minor modifications from the standard method reported by Riccardi and Nicoletti^38^. Briefly, cells were harvested and fixed with 70% cold ethanol and then stored at −20 °C until all cells were ready to be stained. To prepare for PI staining, the cells were centrifuged at 400 × *g* for 5 minutes and supernatants discarded. Cells were washed and resuspended in 0.5 ml of PBS, 0.5 ml of DNA extraction buffer was added, and incubated for 5 minutes. Next, cells were pelleted as above, supernatants were removed, and the cells were resuspended in 1 ml of DNA staining solution and incubated for 30 minutes at room temperature. The DNA extraction buffer and the DNA staining solution were prepared as described in ref. 38. PI intensity in the stained cells was measured using a Becton Dickinson FACS Calibur. Cellular debris and doublets were gated out during the analysis. Following that, hypodiploid cells with an intensity lower than that of the diploid cells (G1) were counted.

### Immunostaining for flow cytometry

Cleavage of PARP and caspase-3 were detected using primary antibodies specific for the cleaved forms of these proteins [Cleaved PARP (Asp214) (D64E10) Rabbit mAb and Cleaved Caspase-3 (Asp175) (5A1E) Rabbit mAb] using the staining protocol recommended by the manufacturer (Cell Signaling Technology). Briefly, infected or control cells were harvested, washed with 5 ml of PBS, then fixed by resuspending the cell pellet in 4% paraformaldehyde at 37 °C for 10 minutes. The tubes were then chilled on ice for 1 minute and permeabilized by resuspending the cell pellet in ice-cold 90% methanol for 30 minutes. Cells were then washed twice with the incubation buffer (1% FBS in PBS) and incubated for 1 hour at room temperature in the presence of the recommended dilution of primary antibody (1:800 for both). Next, the cells were washed with 3 ml of incubation buffer, then incubated for 30 minutes with Alex Fluor^®^ 647 anti-rabbit secondary antibody (Cell Signaling Technology) diluted at 1:400. Cells are then washed and resuspended in 0.5 ml of PBS, then analyzed by flow cytometry using the Becton Dickinson FACS Calibur.

### Caspase and NLRP3 inhibitors

All caspase inhibitors were dissolved in DMSO according to the manufaturer’s instructions (R&D Systems). The inhibitors used were Z-VAD-FMK (pan-caspase inhibitor), Z-IETD-FMK (caspase-8 inhibitor), Z-DEVD-FMK (caspase-3 inhibitor) and Z-WEHD-FMK (caspase-1 inhibitor). The NLRP3 inhibitor (MCC950; Cayman Chemical) was dissolved in DMSO to give a stock solution of 0.5 mg/ml. A working stock of 0.1 μM was prepared fresh in complete medium on the day of the experiment. To ensure efficient inhibition over the four day experiment, at 48 hours p.i., medium was removed and replaced with medium containing freshly prepared inhibitors.

### Measurement of active caspase-1

To measure the levels of active caspase-1, FAM-FLICA^TM^ Caspase-1 Assay Kits were used (ImmunoChemistry Technologies). Briefly, Huh-7.5 cells were seeded in 6-well plates (150,000 cells/well) and infected at an MOI of 1, or uninfected. Four days later, the cells were harvested by trypsinization and stained with FAM-YVAD-FMK according to the manufacturer’s instructions. In co-culture experiments where GFP was included, the FLICA^®^ 660 Caspase-1 Assay Kit, which employs a far-red fluorescent caspase-1 inhibitor, was used.

### LDH assay

Huh-7.5 cells were seeded in 10 cm dishes at 1 × 10^6^ cells/dish and infected on the next day at an MOI of 1, or uninfected. Four days later, 1 ml of the culture medium was collected and clarified. LDH activity was measured using the Pierce^TM^ LDH Cytotoxicity Assay Kit (ThermoFisher Scientific) according to the manufacturer’s instructions.

### Co-culture assay

On the day before initiation of co-culture, 293 T or S29 cells (both non-permissive to HCV) were seeded in antibiotic-free medium at 2 × 10^5^ cells/well in a 24-well plate. In parallel, 10 cm dishes were prepared each containing 1 × 10^6^ Huh-7.5 cells (permissive). Next day, non-permissive cells were transfected with a GFP expression plasmid using Lipofectamine 2000 reagent (Invitrogen) and incubated for 4 hours. In parallel, permissive Huh-7.5 cells were infected with virus at an MOI of 1 and incubated for 3 hours. Immediately following transfection/infection, GFP-transfected non-permissive S29/293 T cells were trypsinized, washed thoroughly with complete medium, then combined with infected or uninfected Huh-7.5 cells at a ratio of 1:5 (S29/293 T:Huh-7.5). After four days of co-culture, cells were harvested and stained with cleaved PARP-specific antibody, cleaved caspase-3-specific antibody, or FAM-YVAD-FMK as described above.

To analyze contact-dependence of HCV-induced cell death, co-culture experiments were performed in 10 cm transwell plates containing inserts with a diameter of 7.5 cm and pore size of 0.4 μm (Corning). Huh-7.5 cells were seeded at 1 × 10^6^ cells/dish and 2 × 10^5^ S29 cells were placed in the insert. Huh-7.5 cells were infected at an MOI of 1 and then the S29-containing insert was placed in the dish. The cells were incubated for four days, then the S29 cells were harvested and stained with either cleaved caspase-3-specific antibody or FAM-YVAD-FMK as described above.

### Guidelines and regulations

No human subjects or human tissue were used in this study, therefore no human consent was required. Cell lines were obtained commercially or from published sources and were used in accordance with all relevant guidelines and regulations. Experiments were performed under BSL2 conditions and were approved by the Memorial University Institutional Biosafety Committee (Biosafety Certificate #M-112-0708).

### Statistical analysis

The data were expressed as the mean +/− the standard deviation (SD). Statistical significance was assessed using the paired Student’s t-test. A *p* value of less than 0.05 was considered significant.

## Additional Information

**How to cite this article**: Kofahi, H. M. *et al*. Hepatitis C Virus Infection of Cultured Human Hepatoma Cells Causes Apoptosis and Pyroptosis in Both Infected and Bystander Cells. *Sci. Rep.* **6**, 37433; doi: 10.1038/srep37433 (2016).

**Publisher's note:** Springer Nature remains neutral with regard to jurisdictional claims in published maps and institutional affiliations.

## References

[b1] Mohd Hanafiah K., Groeger J., Flaxman A. D. & Wiersma S. T. Global epidemiology of hepatitis C virus infection: new estimates of age-specific antibody to HCV seroprevalence. Hepatology 57, 1333–1342, doi: 10.1002/hep.26141 (2013). 23172780

[b2] Freeman A. J. . Estimating progression to cirrhosis in chronic hepatitis C virus infection. Hepatology 34, 809–816, doi: 10.1053/jhep.2001.27831 (2001). 11584380

[b3] Marcellin P. Hepatitis C: the clinical spectrum of the disease. Journal of hepatology 31 Suppl 1, 9–16 (1999). 10622554 10.1016/s0168-8278(99)80368-7

[b4] Schleich K., Krammer P. H. & Lavrik I. N. The chains of death: a new view on caspase-8 activation at the DISC. Cell cycle 12, 193–194, doi: 10.4161/cc.23464 (2013). 23287476 PMC3575441

[b5] Elmore S. Apoptosis: a review of programmed cell death. Toxicologic pathology 35, 495–516, doi: 10.1080/01926230701320337 (2007). 17562483 PMC2117903

[b6] Luo X., Budihardjo I., Zou H., Slaughter C. & Wang X. Bid, a Bcl2 interacting protein, mediates cytochrome c release from mitochondria in response to activation of cell surface death receptors. Cell 94, 481–490 (1998). 9727491 10.1016/s0092-8674(00)81589-5

[b7] Green D. R. Apoptotic pathways: ten minutes to dead. Cell 121, 671–674, doi: 10.1016/j.cell.2005.05.019 (2005). 15935754

[b8] Moldoveanu T., Follis A. V., Kriwacki R. W. & Green D. R. Many players in BCL-2 family affairs. Trends in biochemical sciences 39, 101–111, doi: 10.1016/j.tibs.2013.12.006 (2014). 24503222 PMC4005919

[b9] Riedl S. J. & Salvesen G. S. The apoptosome: signalling platform of cell death. Nature reviews. Molecular cell biology 8, 405–413, doi: 10.1038/nrm2153 (2007). 17377525

[b10] Kerr J. F., Winterford C. M. & Harmon B. V. Apoptosis. Its significance in cancer and cancer therapy. Cancer 73, 2013–2026 (1994). 8156506 10.1002/1097-0142(19940415)73:8<2013::aid-cncr2820730802>3.0.co;2-j

[b11] Saraste A. Morphologic criteria and detection of apoptosis. Herz 24, 189–195 (1999). 10412642 10.1007/BF03044961

[b12] Cookson B. T. & Brennan M. A. Pro-inflammatory programmed cell death. Trends in microbiology 9, 113–114 (2001). 11303500 10.1016/s0966-842x(00)01936-3

[b13] Lamkanfi M. & Dixit V. M. Mechanisms and functions of inflammasomes. Cell 157, 1013–1022, doi: 10.1016/j.cell.2014.04.007 (2014). 24855941

[b14] Schroder K. & Tschopp J. The inflammasomes. Cell 140, 821–832, doi: 10.1016/j.cell.2010.01.040 (2010). 20303873

[b15] Martinon F., Burns K. & Tschopp J. The inflammasome: a molecular platform triggering activation of inflammatory caspases and processing of proIL-beta. Molecular cell 10, 417–426 (2002). 12191486 10.1016/s1097-2765(02)00599-3

[b16] Fink S. L. & Cookson B. T. Caspase-1-dependent pore formation during pyroptosis leads to osmotic lysis of infected host macrophages. Cellular microbiology 8, 1812–1825, doi: 10.1111/j.1462-5822.2006.00751.x (2006). 16824040

[b17] Zhan S. S. . Phagocytosis of apoptotic bodies by hepatic stellate cells induces NADPH oxidase and is associated with liver fibrosis *in vivo*. Hepatology 43, 435–443, doi: 10.1002/hep.21093 (2006). 16496318

[b18] Canbay A. . Apoptotic body engulfment by a human stellate cell line is profibrogenic. Laboratory investigation; a journal of technical methods and pathology 83, 655–663 (2003). 12746475 10.1097/01.lab.0000069036.63405.5c

[b19] Fadok V. A. . Macrophages that have ingested apoptotic cells *in vitro* inhibit proinflammatory cytokine production through autocrine/paracrine mechanisms involving TGF-beta, PGE2, and PAF. The Journal of clinical investigation 101, 890–898, doi: 10.1172/JCI1112 (1998). 9466984 PMC508637

[b20] Jiang J. X., Mikami K., Venugopal S., Li Y. & Torok N. J. Apoptotic body engulfment by hepatic stellate cells promotes their survival by the JAK/STAT and Akt/NF-kappaB-dependent pathways. Journal of hepatology 51, 139–148, doi: S0168-8278(09)00248-7. 19457567 10.1016/j.jhep.2009.03.024 PMC2765371

[b21] Kaimori A. . Transforming growth factor-beta1 induces an epithelial-to-mesenchymal transition state in mouse hepatocytes *in vitro*. The Journal of biological chemistry 282, 22089–22101, doi: 10.1074/jbc.M700998200 (2007). 17513865

[b22] van Zijl F. . Epithelial-mesenchymal transition in hepatocellular carcinoma. Future Oncol 5, 1169–1179, doi: 10.2217/fon.09.91 (2009). 19852728 PMC2963061

[b23] Kubes P. & Mehal W. Z. Sterile inflammation in the liver. Gastroenterology 143, 1158–1172, doi: 10.1053/j.gastro.2012.09.008 (2012). 22982943

[b24] Bartsch H. & Nair J. Chronic inflammation and oxidative stress in the genesis and perpetuation of cancer: role of lipid peroxidation, DNA damage, and repair. Langenbeck’s archives of surgery / Deutsche Gesellschaft fur Chirurgie 391, 499–510, doi: 10.1007/s00423-006-0073-1 (2006). 16909291

[b25] Luedde T. & Schwabe R. F. NF-kappaB in the liver–linking injury, fibrosis and hepatocellular carcinoma. Nature reviews. Gastroenterology & hepatology 8, 108–118, doi: 10.1038/nrgastro.2010.213 (2011). 21293511 PMC3295539

[b26] Ishiguro S. . Serum aminotransferase level and the risk of hepatocellular carcinoma: a population-based cohort study in Japan. European journal of cancer prevention: the official journal of the European Cancer Prevention Organisation 18, 26–32, doi: 10.1097/CEJ.0b013e3282fa9edd (2009). 19077561

[b27] Kumada T. . Incidence of hepatocellular carcinoma in hepatitis C carriers with normal alanine aminotransferase levels. Journal of hepatology 50, 729–735, doi: 10.1016/j.jhep.2008.11.019 (2009). 19232448

[b28] Shlomai A., de Jong Y. P. & Rice C. M. Virus associated malignancies: the role of viral hepatitis in hepatocellular carcinoma. Seminars in cancer biology 26, 78–88, doi: 10.1016/j.semcancer.2014.01.004 (2014). 24457013 PMC4048791

[b29] Cohen-Naftaly M. & Friedman S. L. Current status of novel antifibrotic therapies in patients with chronic liver disease. Therapeutic advances in gastroenterology 4, 391–417, doi: 10.1177/1756283X11413002 (2011). 22043231 PMC3187682

[b30] Czaja A. J. Hepatic inflammation and progressive liver fibrosis in chronic liver disease. World journal of gastroenterology 20, 2515–2532, doi: 10.3748/wjg.v20.i10.2515 (2014). 24627588 PMC3949261

[b31] Walters K. A. . Genomic analysis reveals a potential role for cell cycle perturbation in HCV-mediated apoptosis of cultured hepatocytes. PLoS pathogens 5, e1000269, doi: 10.1371/journal.ppat.1000269 (2009). 19148281 PMC2613535

[b32] Kannan R. P., Hensley L. L., Evers L. E., Lemon S. M. & McGivern D. R. Hepatitis C virus infection causes cell cycle arrest at the level of initiation of mitosis. Journal of virology 85, 7989–8001, doi: 10.1128/JVI.00280-11 (2011). 21680513 PMC3147967

[b33] Marshall A. . Relation between hepatocyte G1 arrest, impaired hepatic regeneration, and fibrosis in chronic hepatitis C virus infection. Gastroenterology 128, 33–42 (2005). 15633121 10.1053/j.gastro.2004.09.076

[b34] Sarfraz S. . Altered expression of cell cycle and apoptotic proteins in chronic hepatitis C virus infection. BMC microbiology 8, 133, doi: 10.1186/1471-2180-8-133 (2008). 18680610 PMC2518161

[b35] Fink S. L., Bergsbaken T. & Cookson B. T. Anthrax lethal toxin and Salmonella elicit the common cell death pathway of caspase-1-dependent pyroptosis via distinct mechanisms. Proceedings of the National Academy of Sciences of the United States of America 105, 4312–4317, doi: 10.1073/pnas.0707370105 (2008). 18337499 PMC2393760

[b36] Bergsbaken T. & Cookson B. T. Macrophage activation redirects yersinia-infected host cell death from apoptosis to caspase-1-dependent pyroptosis. PLoS pathogens 3, e161, doi: 10.1371/journal.ppat.0030161 (2007). 17983266 PMC2048529

[b37] Nagata S., Nagase H., Kawane K., Mukae N. & Fukuyama H. Degradation of chromosomal DNA during apoptosis. Cell death and differentiation 10, 108–116, doi: 10.1038/sj.cdd.4401161 (2003). 12655299

[b38] Riccardi C. & Nicoletti I. Analysis of apoptosis by propidium iodide staining and flow cytometry. Nature protocols 1, 1458–1461, doi: 10.1038/nprot.2006.238 (2006). 17406435

[b39] Bergsbaken T., Fink S. L. & Cookson B. T. Pyroptosis: host cell death and inflammation. Nature reviews. Microbiology 7, 99–109, doi: 10.1038/nrmicro2070 (2009). 19148178 PMC2910423

[b40] Deng L. . Hepatitis C virus infection induces apoptosis through a Bax-triggered, mitochondrion-mediated, caspase 3-dependent pathway. Journal of virology 82, 10375–10385, doi: JVI.00395-08. 10.1128/JVI.00395-08 PMC2573220 18768989

[b41] Zhu H. . Hepatitis C virus triggers apoptosis of a newly developed hepatoma cell line through antiviral defense system. Gastroenterology 133, 1649–1659, doi: 10.1053/j.gastro.2007.09.017 (2007). 17983809

[b42] Mateu G., Donis R. O., Wakita T., Bukh J. & Grakoui A. Intragenotypic JFH1 based recombinant hepatitis C virus produces high levels of infectious particles but causes increased cell death. Virology 376, 397–407, doi: 10.1016/j.virol.2008.03.027 (2008). 18455749 PMC2492671

[b43] Sekine-Osajima Y. . Development of plaque assays for hepatitis C virus-JFH1 strain and isolation of mutants with enhanced cytopathogenicity and replication capacity. Virology 371, 71–85, doi: 10.1016/j.virol.2007.09.019 (2008). 17949770

[b44] Brennan M. A. & Cookson B. T. Salmonella induces macrophage death by caspase-1-dependent necrosis. Molecular microbiology 38, 31–40 (2000). 11029688 10.1046/j.1365-2958.2000.02103.x

[b45] Rayamajhi M., Zhang Y. & Miao E. A. Detection of pyroptosis by measuring released lactate dehydrogenase activity. Methods in molecular biology 1040, 85–90, doi: 10.1007/978-1-62703-523-1_7 (2013). 23852598 PMC3756820

[b46] Chan F. K., Moriwaki K. & De Rosa M. J. Detection of necrosis by release of lactate dehydrogenase activity. Methods in molecular biology 979, 65–70, doi: 10.1007/978-1-62703-290-2_7 (2013). 23397389 PMC3763497

[b47] Silva M. T. Secondary necrosis: the natural outcome of the complete apoptotic program. FEBS letters 584, 4491–4499, doi: 10.1016/j.febslet.2010.10.046 (2010). 20974143

[b48] Thornberry N. A. . A novel heterodimeric cysteine protease is required for interleukin-1 beta processing in monocytes. Nature 356, 768–774, doi: 10.1038/356768a0 (1992). 1574116

[b49] Coll R. C. . A small-molecule inhibitor of the NLRP3 inflammasome for the treatment of inflammatory diseases. Nature medicine 21, 248–255, doi: 10.1038/nm.3806 (2015). PMC4392179 25686105

[b50] Bigger J. E., Tanigawa M., Zhang M. & Atherton S. S. Murine cytomegalovirus infection causes apoptosis of uninfected retinal cells. Investigative ophthalmology & visual science 41, 2248–2254 (2000). 10892869

[b51] Mo J. . Role of Bax in death of uninfected retinal cells during murine cytomegalovirus retinitis. Investigative ophthalmology & visual science 55, 7137–7146, doi: 10.1167/iovs.14-15404 (2014). 25298417 PMC4224579

[b52] Garg H., Mohl J. & Joshi A. HIV-1 induced bystander apoptosis. Viruses 4, 3020–3043, doi: 10.3390/v4113020 (2012). 23202514 PMC3509682

[b53] Geisbert T. W. . Apoptosis induced *in vitro* and *in vivo* during infection by Ebola and Marburg viruses. Laboratory investigation; a journal of technical methods and pathology 80, 171–186 (2000). 10701687 10.1038/labinvest.3780021

[b54] Geisbert T. W. . Pathogenesis of Ebola hemorrhagic fever in cynomolgus macaques: evidence that dendritic cells are early and sustained targets of infection. The American journal of pathology 163, 2347–2370, doi: 10.1016/S0002-9440(10)63591-2 (2003). 14633608 PMC1892369

[b55] Cox A. L. & Siliciano R. F. HIV: Not-so-innocent bystanders. Nature 505, 492–493, doi: 10.1038/505492a (2014). 24451540 PMC4434588

[b56] Russell R. S. . Advantages of a single-cycle production assay to study cell culture-adaptive mutations of hepatitis C virus. Proceedings of the National Academy of Sciences of the United States of America 105, 4370–4375, doi: 10.1073/pnas.0800422105 (2008). 18334634 PMC2393785

[b57] Evans M. J. . Claudin-1 is a hepatitis C virus co-receptor required for a late step in entry. Nature 446, 801–805, doi: nature05654 17325668 10.1038/nature05654

[b58] Joyce M. A. . HCV induces oxidative and ER stress, and sensitizes infected cells to apoptosis in SCID/Alb-uPA mice. PLoS pathogens 5, e1000291, doi: 10.1371/journal.ppat.1000291 (2009). 19242562 PMC2647842

[b59] Calabrese F. . Liver cell apoptosis in chronic hepatitis C correlates with histological but not biochemical activity or serum HCV-RNA levels. Hepatology 31, 1153–1159, doi: 10.1053/he.2000.7123 (2000). 10796892

[b60] Bantel H. . Caspase activation correlates with the degree of inflammatory liver injury in chronic hepatitis C virus infection. Hepatology 34, 758–767, doi: 10.1053/jhep.2001.28229 (2001). 11584373

[b61] Kato T. . Sequence analysis of hepatitis C virus isolated from a fulminant hepatitis patient. Journal of medical virology 64, 334–339 (2001). 11424123 10.1002/jmv.1055

[b62] Ruggieri A., Murdolo M. & Rapicetta M. Induction of FAS ligand expression in a human hepatoblastoma cell line by HCV core protein. Virus research 97, 103–110 (2003). 14602201 10.1016/j.virusres.2003.08.004

[b63] Iken K., Huang L., Bekele H., Schmidt E. V. & Koziel M. J. Apoptosis of activated CD4+ and CD8+ T cells is enhanced by co-culture with hepatocytes expressing hepatitis C virus (HCV) structural proteins through FasL induction. Virology 346, 363–372, doi: 10.1016/j.virol.2005.11.017 (2006). 16336987 PMC2865190

[b64] Negash A. A. . IL-1beta production through the NLRP3 inflammasome by hepatic macrophages links hepatitis C virus infection with liver inflammation and disease. PLoS pathogens 9, e1003330, doi: 10.1371/journal.ppat.1003330 (2013). 23633957 PMC3635973

[b65] Shrivastava S., Mukherjee A., Ray R. & Ray R. B. Hepatitis C virus induces interleukin-1beta (IL-1beta)/IL-18 in circulatory and resident liver macrophages. Journal of virology 87, 12284–12290, doi: 10.1128/JVI.01962-13 (2013). 24006444 PMC3807883

[b66] Chen W. . HCV genomic RNA activates the NLRP3 inflammasome in human myeloid cells. PloS one 9, e84953, doi: 10.1371/journal.pone.0084953 (2014). 24400125 PMC3882267

[b67] Chen G. Y. & Nunez G. Sterile inflammation: sensing and reacting to damage. Nature reviews. Immunology 10, 826–837, doi: 10.1038/nri2873 (2010). PMC3114424 21088683

[b68] Geng Y. . Heatstroke induces liver injury via IL-1beta and HMGB1-induced pyroptosis. Journal of hepatology 63, 622–633, doi: 10.1016/j.jhep.2015.04.010 (2015). 25931416

[b69] Antonopoulos C. . Caspase-8 as an Effector and Regulator of NLRP3 Inflammasome Signaling. The Journal of biological chemistry 290, 20167–20184, doi: 10.1074/jbc.M115.652321 (2015). 26100631 PMC4536427

[b70] Sagulenko V. . AIM2 and NLRP3 inflammasomes activate both apoptotic and pyroptotic death pathways via ASC. Cell death and differentiation 20, 1149–1160, doi: 10.1038/cdd.2013.37 (2013). 23645208 PMC3741496

[b71] Chung H. . NLRP3 regulates a non-canonical platform for caspase-8 activation during epithelial cell apoptosis. Cell death and differentiation, 23, 1331–1346, doi: 10.1038/cdd.2016.14 (2016). 26891693 PMC4947664

[b72] Vajjhala P. R. . The Inflammasome Adaptor ASC Induces Procaspase-8 Death Effector Domain Filaments. The Journal of biological chemistry 290, 29217–29230, doi: 10.1074/jbc.M115.687731 (2015). 26468282 PMC4705927

[b73] Chen M. . Internalized Cryptococcus neoformans Activates the Canonical Caspase-1 and the Noncanonical Caspase-8 Inflammasomes. Journal of immunology 195, 4962–4972, doi: 10.4049/jimmunol.1500865 (2015). 26466953

[b74] Lebeaupin C. . ER stress induces NLRP3 inflammasome activation and hepatocyte death. Cell death & disease 6, e1879, doi: 10.1038/cddis.2015.248 (2015). 26355342 PMC4650444

[b75] Upton J. W. & Chan F. K. Staying alive: cell death in antiviral immunity. Molecular cell 54, 273–280, doi: 10.1016/j.molcel.2014.01.027 (2014). 24766891 PMC4010939

[b76] Fischer R., Baumert T. & Blum H. E. Hepatitis C virus infection and apoptosis. World journal of gastroenterology 13, 4865–4872 (2007). 17828818 10.3748/wjg.v13.i36.4865 PMC4611765

[b77] Bantel H. & Schulze-Osthoff K. Apoptosis in hepatitis C virus infection. Cell death and differentiation 10 Suppl 1, S48–58, doi: 10.1038/sj.cdd.4401119 (2003). 12655346

[b78] Blight K. J., McKeating J. A. & Rice C. M. Highly permissive cell lines for subgenomic and genomic hepatitis C virus RNA replication. Journal of virology 76, 13001–13014 (2002). 12438626 10.1128/JVI.76.24.13001-13014.2002 PMC136668

[b79] Jones D. M., Atoom A. M., Zhang X., Kottilil S. & Russell R. S. A genetic interaction between the core and NS3 proteins of hepatitis C virus is essential for production of infectious virus. Journal of virology 85, 12351–12361, doi: 10.1128/JVI.05313-11 (2011). 21957313 PMC3209351

[b80] Zhong J. . Robust hepatitis C virus infection *in vitro*. Proceedings of the National Academy of Sciences of the United States of America 102, 9294–9299, doi: 10.1073/pnas.0503596102 (2005). 15939869 PMC1166622

